# When deadman theory meets footprint decortication: a suture anchor biomechanical study

**DOI:** 10.1186/s13018-019-1209-7

**Published:** 2019-05-27

**Authors:** Chih-Kai Hong, Kai-Lan Hsu, Fa-Chuan Kuan, Ping-Hui Wang, Che-Chia Hsu, Ming-Long Yeh, Wei-Ren Su

**Affiliations:** 1 Department of Orthopaedic Surgery, National Cheng Kung University Hospital, College of Medicine, National Cheng Kung University, No.138, Sheng-Li Road, Tainan City, 70428 Taiwan; 2 Department of Biomedical Engineering, National Cheng Kung University, Tainan, Taiwan; 3 Department of Orthopedics, Chi-Mei Medical Center, Tainan, Taiwan; 4 Medical Device Innovation Center, National Cheng Kung University, Tainan, Taiwan; 5 Medical Device R & D Core Laboratory, National Cheng Kung University Hospital, Tainan, Taiwan

**Keywords:** Suture anchor, Insertion angle, Decortication, Biomechanics

## Abstract

**Background:**

The optimal insertion angle for suture anchor insertion has long been of great interest. Although greater tuberosity decortication is commonly performed during rotator cuff repair, the effect of decortication on the suture anchor insertion angle remains unclear. The purpose of this study was to compare the pullout strength of threaded suture anchors inserted at 45° and 90° in decorticated and non-decorticated synthetic bone models.

**Methods:**

Two kinds of synthetic bones were used to simulate the decorticated and non-decorticated conditions, for which 40 metallic suture anchors were used. Anchors were inserted at 45° and 90° in both decorticated and non-decorticated models and tested under cyclic loading followed by load-to-failure testing. The number of completed cycles, ultimate failure load, and failure modes was recorded.

**Results:**

In the decorticated model, the ultimate failure load of anchors inserted at 45° (67.5 ± 5.3 N) was significantly lower than that of anchors inserted at 90° (114.1 ± 9.8 N) (*p* <  0.001). In the non-decorticated model, the ultimate failure load of anchors inserted at 45° (591.8 ± 58 N) was also significantly lower than that of anchors inserted at 90° (724.9 ± 94 N) (*p* = 0.003). Due to the diverse failure modes in the non-decorticated model, specimens with a failure mode of suture anchor pullout were analyzed in greater detail, with results showing a significantly larger pullout strength for anchors inserted at 90° (781.6 ± 53 N) than anchors inserted at 45° (648.0 ± 43 N) (*p* = 0.025).

**Conclusion:**

Regardless of decortication, the pullout strength of anchors inserted at 90° was greater than those inserted at 45°. The clinical relevance is that inserting suture anchors at 90° is recommended due to the significantly larger ultimate failure load in both decorticated and non-decorticated bones.

## Introduction

Techniques involving suture anchors have become prevalent for securing soft tissues to the bone in different parts of the body [1–5], the most common site of which is the shoulder [6–8]. Although various suture anchors have been developed and evaluated [9, 10], their fixation strengths are affected by several factors, including the design, bone density, insertion depth, and insertion angle [6, 7, 9–13]. In 1995, Burkhart introduced the “deadman” theory, which concerns the trigonometric calculation of the suture-anchor insertion angle [14]. Since then, a number of biomechanical studies have tested the theory [6, 7, 15–19]; however, results have been inconsistent, leaving the optimal suture-anchor insertion angle open to debate. Itoi et al. comprehensively evaluated their laboratory data against previous studies and concluded that the insertion angles of 45° and 90° were the strongest for threadless and threaded anchors, respectively [20, 21].

Greater tuberosity (GT) decortication is commonly performed during rotator cuff repair since it may promote biological healing at the bone-tendon junction [22]. Although the ideal preparation procedure remains uncertain, several studies have revealed that footprint preparation had positive effects on tendon-to-bone healing [23–25]. Nevertheless, decortication of the rotator cuff footprint affects the suture-anchor biomechanics [22]. Hyatt et al. compared the pullout strength of suture anchors in non-decorticated and decorticated footprints in a biomechanical study, the results of which showed that the pullout strength of the suture anchor significantly decreased after decortications [22].

Despite the insertion angle of suture anchors having been discussed in various biomechanical models [6, 7, 15–19], whether the insertion-angle strength is affected by decortication remains unclear. The purpose of this study was to compare the pullout strengths of threaded suture anchors inserted at 45° and 90° into decorticated and non-decorticated synthetic bone models. We hypothesized that threaded anchors inserted at 90° would have greater pullout strength than those inserted at 45° for both the non-decorticated and decorticated synthetic bone models.

## Materials and methods

### Experiment subjects

Two kinds of synthetic bones (Sawbones, Pacific Research Laboratories, Vashon, WA) were used in this study. To simulate the decorticated bone, solid rigid polyurethane foam with a density of 0.16 g/cm^3^ was cut into blocks measuring 60 mm in width, 42 mm in depth, and 40 mm in height (named decorticated synthetic bone). To simulate the non-decorticated bone, blocks of the same polyurethane foam were laminated with 2 mm of short-fiber-filled epoxy (density of 1.63 g/cm^3^) attached on one side (named non-decorticated synthetic bone). Tingart et al. reported that the volumetric bone mineral density of the humeral GT ranged from 0.10 ± 0.03 to 0.18 ± 0.04 g/cm^3^ in a cadaveric study [26]; accordingly, polyurethane foam with a density of 0.16 g/cm^3^ was selected. In addition, the 2-mm-thick cortical bone was chosen in accordance with a previous study [7].

**Fig. 1:**
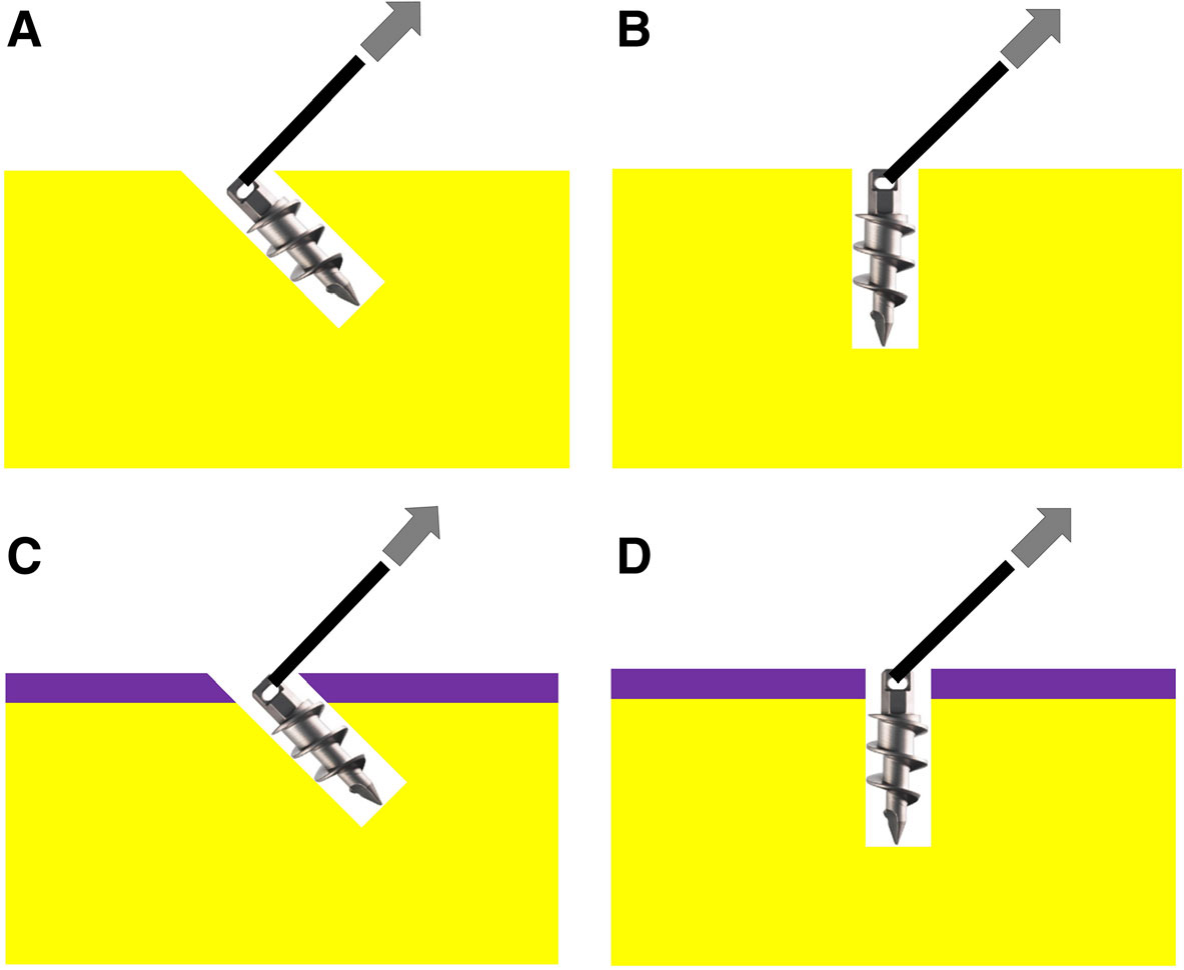
**a** Anchors inserted at 45° to the surface in the decorticated bone model (D-45 group). **b** Anchors inserted at 90° to the surface in the decorticated bone model(D-90 group). **c** Anchors inserted at 45° to the surface in the non-decorticated bone model (N-45 group). **d** Anchors inserted at 90° to the surface in the non-decorticated bone model (N-90 group)

Metallic suture anchors (Super Revo, 5 mm, ConMed Corporation, Utica, NY) with double-loaded no. 2 braided sutures were used for biomechanical testing. As suggested in previous studies [7, 27], the original sutures were replaced with the braided polyethylene lines (No.8 Jigging PE; Amika, Japan) to avoid suture breakage during biomechanical testing. A total of 40 suture anchors were used, with each suture anchor randomly assigned to one of the following four groups: decortication with anchor inserted at 45° (D-45 group), decortication with anchor inserted at 90° (D-90 group), non-decortication with anchor inserted at 45° (N-45 group), and non-decortication with anchor inserted at 90° (N-90 group), as illustrated in Fig. 1. Each suture anchor was inserted into one synthetic bone block; insertion of the suture anchors was performed in accordance with the manufacturers’ instructions. The suture anchors were inserted at 45° to the bone surface in the D-45 and N-45 groups, and at 90° to the bone surface in D-90 and N-90 groups. The top end of the eyelet of the suture anchor was level with the bone.

### Biomechanical testing setup

**Fig. 2:**
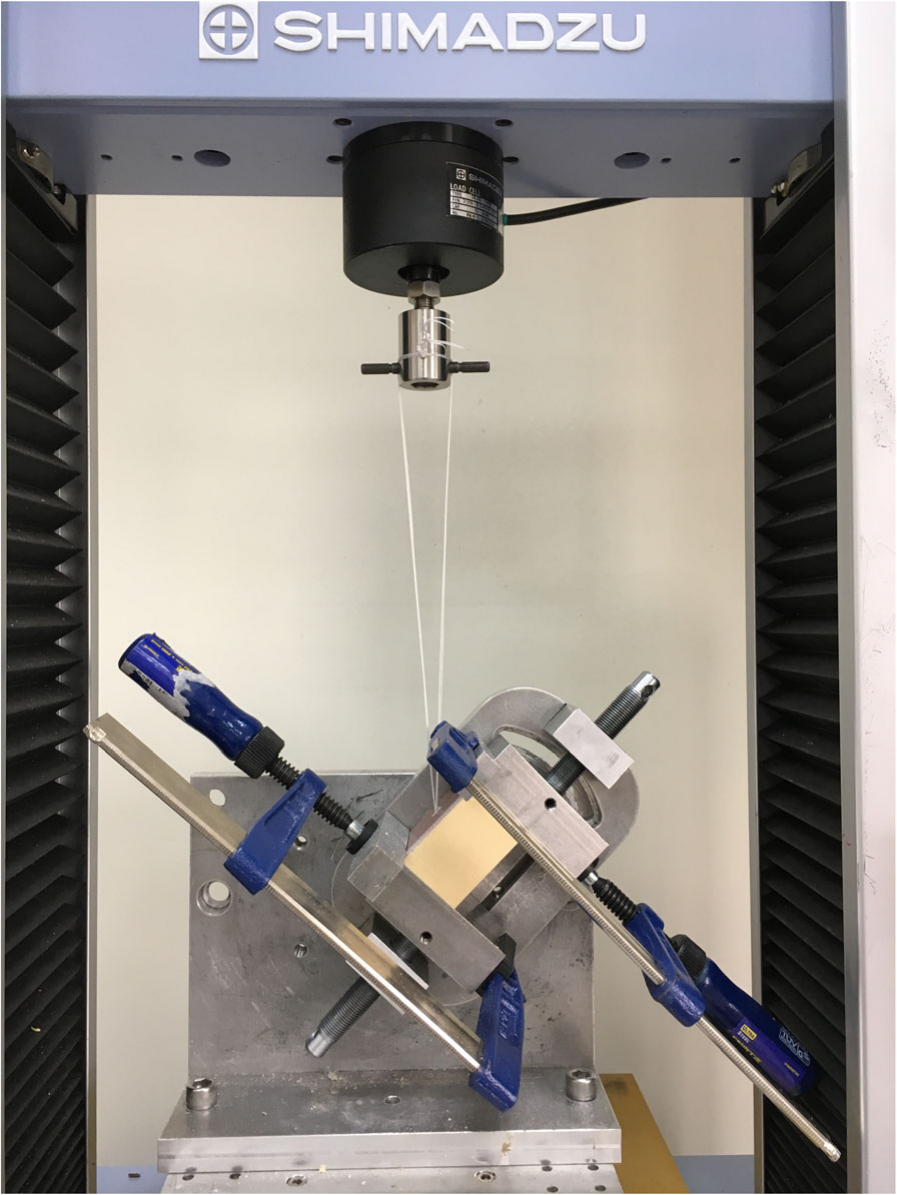
Experimental setup of the biomechanical testing

Each synthetic bone block was mounted on a universal material testing system (AG-X; Shimadzu, Tokyo, Japan). A custom-made clamp was used to fix the synthetic bone block, after which the suture ends were knotted together and looped over a post on the adapter of the material testing machine. The custom-made clamp was rotated to make a 45° angle between the suture and the bone surface (Fig. 2). Sutures were pulled at 45° (load applied 90° to the axis of anchor insertion when the anchor was inserted at 45° to the bone surface) to simulate the physiologic pull of the supraspinatus tendon [7, 20].

### Biomechanical testing protocol

Similar to previous studies [13, 22], each specimen was cyclically loaded at a crosshead speed of 1 mm/s, for which the cyclic loading protocol [22] was as follows: 0 to 50 N for 10 cycles, 0 to 100 N for 100 cycles, 0 to 150 N for 10 cycles, and 0 to 200 N for 10 cycles. After cyclic loading, the specimens were loaded to failure. The number of completed cycles, ultimate loads to failure, and failure modes were recorded.

### Statistical analysis

Statistical comparisons were performed with SPSS for Windows, Version 16 (SPSS Inc., Chicago, IL, USA). Descriptive statistics, including means and standard deviations, were obtained for each group. The Mann-Whitney *U* test was used to compare the ultimate loads to failure for the suture anchors at 45° and 90° in both the decorticated and non-decorticated groups. Statistical significance was set as a *p* value less than 0.05.

### Post hoc power analyses

Post-hoc power analyses were performed with G*Power Version 3.1.3 (Heinrich Heine-University of Dusseldorf, Dusseldorf, Germany) to calculate the achieved power. An alpha equal to 0.05 was given for these models.

## Results

### Decorticated synthetic bone model

All specimens in the D-45 group completed the 0–50 N cyclic loading test, but failed in the first cycle of the 0-100 N cyclic loading test. On the other hand, all specimens in the D-90 group completed the 0–100 N cyclic loading test, but failed in the first cycle of the 0-150 N cyclic loading test. The maximum load to failure in the D-45 group (67.5 ± 5.3 N) was significantly smaller than that in the D-90 group (114.1 ± 9.8 N) (*p* <  0.001). The calculated effect size of the results was 5.94; and with a given α equal to 0.05, the post hoc achieved power was 99.9%. All specimens in the D-45 and D-90 groups failed from suture anchor pullout (Table 1).

**Table 1:** Completed cycles, ultimate failure load, and failure modes in the different groups

Insertion angle	Completed cycles	Ultimate failure load (mean ± SD)	*p* value	Failure modes
Decorticated synthetic bone model				
45°	10	67.5 ± 5.3 N	< 0.001*	10 anchor pullout
90°	20	114.1 ± 9.8 N		10 anchor pullout
Non-decorticated synthetic bone model				
45°	40	591.8 ± 58 N	0.003*	3 anchor pullout 2 suture rupture at eyelet 5 broken eyelet
90°	40	724.9 ± 94 N		5 anchor pullout 5 suture rupture at eyelet

*Significantly different in ultimate failure load between two groups with the Mann-Whitney *U* test (*p* < 0.05)

### Non-decorticated synthetic bone model

All specimens in both the N-45 and N-90 groups completed all cyclic loading tests, and so were able to be subjected to the final load to failure test. The ultimate load to failure in the N-45 group (591.8 ± 58 N) was significantly smaller than that in the N-90 group (724.9 ± 94 N) (*p* = 0.003). The calculated effect size of the results was 1.70, and with a given *α* equal to 0.05, the post hoc achieved power was 93.8%. The failure modes in both groups varied. In the N-45 group, three specimens failed from suture anchor pullout, two failed from suture rupture at the eyelet, and five failed from a broken eyelet. In the N-90 group, five specimens failed from suture anchor pullout and the other five failed from suture rupture at the eyelet (Table 1).

Due to the diverse failure modes in the non-decorticated bone model, specimens with a failure mode of suture anchor pullout (3 in the N-45 group and 5 in the N-90 group) were analyzed in greater detail. The ultimate failure load of these specimens in the N-45 group (648 ± 43 N) was significantly smaller than that of the specimens in the N-90 group (782 ± 53 N) (*p* = 0.025). The calculated effect size for these data was 2.67, and with a given *α* equal to 0.05, the post hoc achieved power was 83.4%.

## Discussion

The principal findings of this study showed that the ultimate loads to failure in both the decorticated and non-decorticated synthetic bone models were significantly higher when the suture anchors were inserted at 90° than at 45°. The optimal insertion angle for suture anchor insertion has been widely discussed in previous biomechanical models [6, 7, 15–18]. Since decortications are commonly performed due to the perceived biological benefit, the current study further investigated whether decortications would affect the optimal suture-anchor insertion angle.

Footprint decortication is a common procedure in rotator cuff repair surgeries since it potentially promotes tendon healing with significantly increased ultimate force to failure and improved microscopic histological findings [23–25]. However, footprint decortications affect suture-anchor biomechanics [22]. Hyatt et al. assessed the pullout force of suture anchors in decorticated and non-decorticated footprints in a cadaveric humeri model. Their results indicated that decortications of the rotator cuff footprint significantly decreased the pullout strength of the suture anchor [22]. Since the effect of decortication on the suture-anchor insertion angle has been rarely discussed, our study further compared the pullout strengths between suture anchors inserted at 45° and 90° in a decortication model. Our results showed that the pullout strength of suture anchors in decorticated synthetic bone was significantly larger when inserted at 90°. Consequently, an insertion angle of 90° is recommended when inserting a suture anchor after footprint decortication.

The strength of the anchor material is higher than that of bone, and so from observations of the biomechanical testing, it appears that the fracture mechanism of anchor pullout is attributable to the “anchor thread” or the “anchor itself” cutting through the bone. The reason that anchors inserted in decorticated bones at 90° showed greater pullout strength compared to 45°may be explained by the force diversion of the applied traction force causing a difference in stress distribution around the bones, as well as the different vertical depths of the anchors in bones, between the groups. Sano et al. reported in a finite element study that there was higher stress around the proximal anchor threads on the traction side [17]; further, the maximum value of the equivalent stress on the bone was greater for the 45°insertion setting than for the 90°insertion setting when the traction force was applied at 45°to the bone surface [17]. Adding to this, we observed a phenomenon whereby anchors rotated before being pulled out during the biomechanical testing for the anchors inserted at 45°, with the rotation centers located near the end of the anchors. As such, the anchors protruded from the bone surface after rotation, which meant their depths became shallower, resulting in less constriction force between the anchors and the bone. Consequently, the pullout strength of anchors inserted at 45°was smaller than those inserted at 90°.

Compared to the effect of the different insertion angle, the effect of decortication on the maximal pullout strength was more significant. In the present study, the ultimate failure loads in non-decorticated bones (592 N and 725 N for insertion angle of 45° and 90° respectively) were much higher than those in decorticated bones (68 N and 114 N for insertion angle of 45° and 90° respectively). Thus, in clinical practice, surgeons should also pay attention to the effect of decortication.

Although previous studies have evaluated the pullout strength of suture anchors in a model with synthetic bone blocks that had similar properties to cancellous bone [6, 15], there were certain shortcomings in their biomechanical testing setups. According to the statements from Itoi et al., the angle of applied load to a suture anchor would be 45° to the insertion surface [20]. Itoi et al. reasoned that when the tendon is pulled medially, the suture passing through the tendon would incline until the retraction force and the horizontal component of the force through the suture reached equilibrium [20]; as a result, the angle of applied load to a suture anchor would be 45° to the insertion surface, and so surgeons do not have to worry about this angle in surgeries [20]. In a study by Clevenger et al. [6], however, the applied load to a suture anchor was reported to be 0° to the insertion surface, not 45°. Similarly, although multiple pulling angles on suture anchors were evaluated by Green et al. [15], the pulling angle of 45° to the insertion surface was not assessed. In response to these issues, the present study investigated the pullout strength of suture anchors with a force pulled at 45° to the insertion surface in a similar biomechanical model. Accordingly, the model in this study could better simulate the physiological pull of the supraspinatus tendon, and so we believe our findings should be more representative.

The current study demonstrated that the ultimate load to failure was significantly higher when the suture anchors were inserted at 90° compared to 45° in the non-decorticated synthetic bone model, which is consistent with previous research [7]. Nagamoto et al. [7] reported respective pullout strengths of 711.4 ± 25.3 N and 599.2 ± 29.8 N when suture anchors were inserted into medium-density bones at 90° and 45° and pulled at 45°. Similarly, the present study recorded the ultimate failure loads of 782 ± 53 N and 648 ± 43 N when the suture anchors were inserted at 90° and 45°, respectively. Since a variety of failure modes were found in the non-decorticated synthetic bone model in the current study, we analyzed the failure-mode data of suture anchor pullout in greater detail; in this manner, we could better evaluate the “pullout strength” of the suture anchors. Not surprisingly, our results showed that anchors inserted at 90° had significantly greater pullout strength than those inserted at 45°.

In addition to biomechanical studies, the optimum insertion angle of a suture anchor has also been intensively discussed. Dr. Burkhart has addressed several comments on this topic, with one of the flaws pointed out being that the eyelets of the suture anchors were not at or below the surface level of the bone [28]. In response to this point, we ensured all suture anchors were inserted sufficiently deep that the anchor eyelets were at bone level in the present study.

It is worth noting that the optimal insertion angles for threaded and threadless anchor were different. Both the previous study [7] and our study suggested that threaded anchors inserted at 90° had a significantly higher ultimate load to failure than those inserted at 45°. In contrast, Nagamoto et al. reported that the pullout strength of threadless anchor inserted at 45° to the bone surface was the strongest followed by 90° and the weakest at 135° [21]. Therefore, the selection of optimal insertion angle should also depend on the use of threaded or threadless anchors.

Although anchor pullout was the most common failure load in our study, it may not occur often in non-decorticated bones clinically since the suture materials were replaced. According to the previous studies [29, 30] and our preliminary data, the suture-rupture load of double-loaded no. 2 polyethylene braided sutures was around 550–650 N. In this study, the ultimate failure load in the non-decorticated bone model could reach 725 N after replacing the sutures materials. The aforementioned findings suggested that suture rupture would occur before anchor pullout when inserting suture anchors in non-decorticated bones in real clinical condition.

### Limitations

There are some limitations in this study. First, the current study used synthetic bones for biomechanical testing, rather than cadaveric humeri. Nevertheless, this biomechanical model has been applied in several studies [6, 7, 15]. Second, the suture material used was different from that typically employed in clinical use. Using alternative sutures in biomechanical studies has been recommended since preliminary testing has demonstrated that original sutures are too weak to complete biomechanical testing [7, 22, 27]. Be that as it may, we believe that switching suture materials may not be relevant because we target only the bone-to-anchor interface. Third, this study exclusively evaluated the pullout strength of the anchor itself. As such, future studies may be required to evaluate the pullout strength after tendon repair. Fourth, only metal screws were used in this study; however, non-metallic screws are becoming increasingly popular. Therefore, findings from the current study may not represent conditions in which non-metallic screws are employed.

## Conclusion

Regardless of decortication, the pullout strength of anchors inserted at 90° was greater than those inserted at 45°.

## Data Availability

The datasets used and/or analyzed during the current study are available from the corresponding author on reasonable request.

## Abbreviation

GT: Greater tuberosity
